# Acquired Von Willebrand Deficiency in Adults With Aortic Stenosis: A Systematic Review

**DOI:** 10.7759/cureus.28879

**Published:** 2022-09-07

**Authors:** Prasana Ramesh, Suthasenthuran Kanagalingam, FNU Zargham Ul Haq, Nishok Victory Srinivasan, Aujala Irfan Khan, Ghadi D Mashat, Mohammad Hazique, Kokab Irfan Khan, Safeera Khan

**Affiliations:** 1 Internal Medicine, California Institute of Behavioral Neurosciences & Psychology, Fairfield, USA; 2 General Surgery, California Institute of Behavioral Neurosciences & Psychology, Fairfield, USA; 3 Research, California Institute of Behavioral Neurosciences & Psychology, Fairfield, USA; 4 Pediatrics, California Institute of Behavioral Neurosciences & Psychology, Fairfield, USA

**Keywords:** prisma, bleeding, a systematic review, aortic stenosis (as), von-willebrand factor

## Abstract

Von Willebrand factor (VWF) deficiency is associated with bleeding complications. The congenital type of Von Willebrand disease(VWD) is a very well-known bleeding disorder and sometimes may be associated with life-threatening hemorrhage. This systematic review is aimed at gathering further knowledge regarding the pathology of an acquired VWD form within a population of patients with aortic stenosis (AS) by shortlisting quality articles on this theme, through the Preferred Reporting Items for Systematic Review and Meta-analysis (PRISMA) 2020 guidelines. High shear stress caused by the stenotic valve cleaves VWF multimers, causing a relative state of deficiency. The condition returns to baseline immediately following surgical replacement of the valve. Results across eight studies reviewed by a majority concluded that in an AS patient with bleeding, the most likely cause is an acquired deficiency of VWF, associated with factors influencing blood flow and caused by the in-situ valve. However, several studies suggested otherwise/were misclassifications. This review highlighted the relationship between AS and acquired VWF deficiency and should be foreseen as an adverse complication, attracting further research and future theragnostic strategies for this condition.

## Introduction and background

The prevalence of aortic stenosis (AS) varies among high-income and low-income countries. AS is the second most common valvular lesion in the United States [1]. While calcific AS accounts for most cases in developed countries, Rheumatic Heart Disease is a major cause of this lesion within developing and underdeveloped nations [1]. Heyde syndrome is the association between gastrointestinal (GI) bleeding from an arteriovenous malformation (AVM) and AS [2]. Von Willebrand factor (VWF) is a hemostatic plasma glycoprotein. It binds to Factor VIII (FVIII), platelet surface glycoproteins, and connective tissue. Therefore, it stabilizes FVIII within the circulation [3]. The largest VWF multimers are typically contained within storage granules.

Ultra-large VWF are rapidly removed from the plasma of healthy individuals. This is because they are pro-thrombotic and cause spontaneous platelet aggregation. ADAMTS-13 cleaves VWF into two smaller pieces, which regulates the plasma level of VWF [1]. The shear stress undergone by the VWF can lead to its proteolysis. When conformational changes occur in ultra-large VWF, they are cleaved by the enzymes [3]. High molecular weight multimer deficiency - caused by increased shear stress due to the stenosed valve - is believed to contribute to VWF deficiency in AS [4]. Due to conflicting evidence within previous literature and limited awareness amongst the general population and physicians, it is important to review the association between the above [5].

This systematic review probed published articles on the association between AS and acquired von-Willebrand disease (AVWD). The findings were endorsed to report the simultaneous incidence and suggest a true relationship between AS and AVWD.

## Review

Methods

This review employed the Preferred Reporting Items for Systematic Reviews and Meta-Analyses (PRISMA) 2020 [6]. Protocol details for this systematic review are described below.

Search Sources and Strategy

PubMed, PubMed Central (PMC), Science Direct, Cochrane Library, and Google Scholar were searched for literature published after 2000. The databases were explored using three keywords: ‘aortic stenosis’, ‘acquired Von Willebrand disease, and ‘anemia’. The keywords were used separately and consequently combined using Boolean "AND". Within Google Scholar, this strategy was employed to identify published articles. Within PubMed and PMC, keywords and their concept identification words were combined using Boolean "OR", entered in order to create a Medical Subject Heading (MeSH) search strategy. All MeSH strategies were combined using Boolean "AND". The final MeSH keyword entered was the following: "Aortic stenosis ("Aortic Valve Stenosis/complications"[MeSH] OR 

"Aortic Valve Stenosis/etiology"[MeSH] OR "Aortic Valve Stenosis/physiopathology"[MeSH]) AND “Von Willebrand disease ("von Willebrand Diseases/classification"[Majr] OR 

"von Willebrand Diseases/diagnosis"[Majr] OR "von Willebrand Diseases/etiology"[Majr] OR 

"von Willebrand Diseases/genetics"[Majr] OR "von Willebrand Diseases/physiopathology"[Majr])”

Eligibility Criteria

Duplicate articles were screened and removed. Following reading the title and abstract, irrelevant articles were removed. The selected papers were subjected to a quality appraisal, and only those that met > 60% of the assessment criteria were selected. Only articles published between 2001 and 2021 were included among the selected articles. Articles published in English and focusing on the adult population (18-80 years old) were included. Articles including pediatric populations and unpublished literature/grey literature were excluded.

Quality Assessment

The JBI Manual for evidence synthesis was used for quality appraisal of the case reports [7]. Observational studies were subjected to quality appraisal using the Modified Newcastle-Ottawa Scale [8]. The different components were formulated in the table. Each published observational study selected for quality appraisal was extensively subjected to assessment with various components like QE (Quality of Evidence), REC (Representativeness of the exposed cohort), SNEC (Selection of the non-exposed cohort), AE (Ascertainment of exposure), DEMO (Demonstration that the outcome of interest was not present at the start of the study), CC (Comparability of cohorts [based on design or analysis]), AO (Assessment of outcome), FUDS (Follow-up duration sufficiency for outcomes to occur), AFC (Adequacy of follow-up of cohorts). A minimum score of 7 and above was considered a high-quality study.

**Figure 1:**
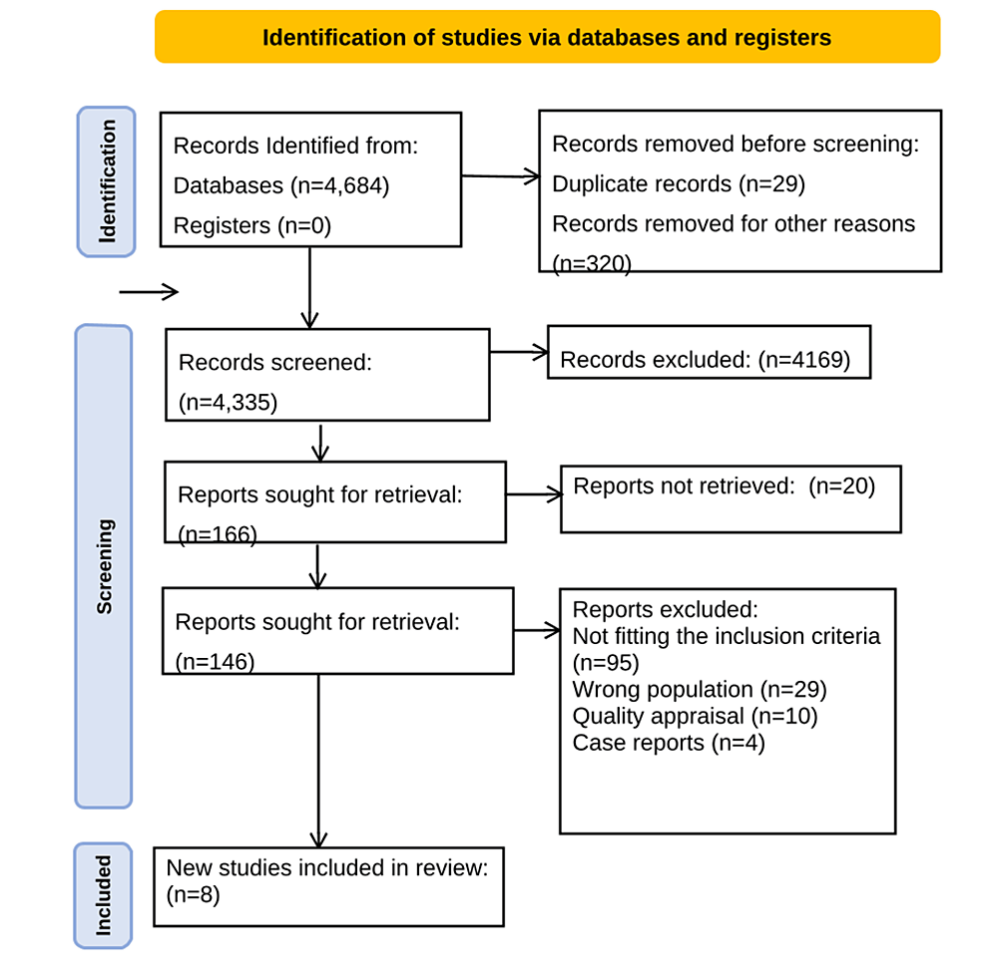
Preferred Reporting Items for Systematic Reviews and Meta-Analyses (PRISMA) flow diagram employed for this systematic review. This figure was created by Prasana Ramesh

The PRISMA flow diagram depicting this review’s search methodology is shown in Figure 1.

Results

A total of 4,684 articles were identified from PubMed, PubMed Central (PMC), Science Direct, Cochrane Library, and Google Scholar by titles and abstracts. The articles were checked for duplication using the EndNote tool (Clarivate, Philadelphia, USA). 29 duplicates were removed. 320 articles were totally irrelevant and therefore were removed. After removing articles for sporadic reasons, 166 out of 4,335 articles were selected. They were subjected to full-text review. The information about 20 articles could not be retrieved. 95 articles did not meet the inclusion criteria, and 29 articles involved the wrong population.

10 articles did not meet the minimum TSC set during the quality appraisal check. Six of them scored five and the remaining scored less than 5. Case reports that did not meet eligibility while using the JBI checklist. This led to a total of 17 articles which were consequently subjected to a quality appraisal. 17 articles were subjected to a quality appraisal. Eight high-quality articles were selected following the final review and quality appraisal. This included two case reports which satisfied the quality appraisal screened using the JBI Manual for evidence synthesis [7].

Quality appraisal of observational studies through the Modified Newcastle-Ottawa Scale is shown in Table 1 [8].

**Table 1:** Modified New-Castle Ottawa Scale for observational studies. Key - #: one point given to a study if it fulfills criteria (blank = nil) TSC: Total Score (max. score = 9); QE: Quality of Evidence; REC: Representativeness of the exposed cohort; SNEC: Selection of the non-exposed cohort; AE: Ascertainment of exposure; DEMO: Demonstration that the outcome of interest was not present at the start of the study; CC: Comparability of cohorts (based on design or analysis); AO: Assessment of outcome; FUDS: Follow-up duration sufficiency for outcomes to occur; AFC: Adequacy of follow-up of cohorts.

Study	Selection*				Comparability		Outcome***			TSC	QE
	REC	SNEC	AE	DEMO	CC	Comparable ?	AO	FUDS	AFC		
Bolliger et al. [9]	#		#	#	#		#	#	#	7	High
Casonato et al. [10]	#	#	#	#	#		#	#	#	8	High
Bender et al. [11]	#		#	#	#		#	#	#	8	High
Nagao et al. [12]	#	#		#	#		#	#	#	7	High
Kellermair et al. [4]	#	#		#	#		#	#	#	7	High
Casonato et al. [13]	#	#	#	#	#		#	#	#	8	High

Features and outcomes of studies included in this review are highlighted in Table 2. 

**Table 2:** Table of features and outcomes of studies included in the systematic review. AS: Aortic Stenosis; AVR: Aortic Valve Replacement; VWF: Von Willebrand Factor; HMWVWF: High Molecular weight multimers of Von Willebrand Factor; AVWS: Acquired Von Willebrand Syndrome.

No.	Author	Year	Country	Type of Study	Population	Sample Size	Follow-up Duration	Outcomes
1.	Bolliger et al. [9]	2011	Switzerland	Cohort	Adults	60		The altered VWF multimer structure pre-AVR was not associated with increased bleeding. These findings are explained by the perioperative release of VWF and rapid recovery of the largest VWF multimers.
2.	Casonato et al. [10]	2011	Italy	Cohort	Adults	41		Within 24 hours of AVR, VWF multimers returned to normal levels and persisted thereafter for 6 months. This indicates a relationship between AS and VWF deficiency.
3.	Bander et al [11]	2012	USA	Cohort	Adults	114		ADAMTS-13 declining post-surgery can account for the acute rise in highest-molecular weight multimers of VWF following AVR. Regression of HMWVWF multimers during the weeks following AVR reflects the resolution of the effects of surgery on ADAMTS-13. The persistent increase in multimers documented six months later indicated the proportion attributable to AS alleviation.
4.	Nagao et al [12]	2018	Italy	cohort	Adults	3403		Patients with severe AS are particularly susceptible to anemia because they often suffer from acquired coagulopathy (von Willebrand syndrome type 2A, which increases the risk of bleeding causing anemia which is a common comorbidity in patients with severe AS.
5.	Kellermair et al [4]	2018	Austria	Case-control	Adults	31		AVWS in AS patients is not similar to those in Type 2A. Therefore, further studies are required to support the evidence and prevent misclassification.
6.	Casonato et al. [13]	2020	Italy	Case-Control	Adults	5		Patients with inherited von Willebrand disease can develop the acquired deficiency in addition. History of bleeding should be given an important consideration as valve replacement may be associated with significant hemorrhage.
7.	Kapila et al. [14]	2013	USA	Case report	Adult	1		Platelet adhesion mediated by VWF multimers for hemostasis undergoes degeneration by high shear stress across the stenotic aortic valve, leading to acquired von Willebrand's disease (Type 2A VWF disease). Surgery alone can result in the cure of this condition (Heyde syndrome).
8.	Iijima et al. [15]	2018	Japan	Case report	Adult	1		The shear stress caused by the stenosed aortic valve causes VWF to stretch. Therefore, it causes it to be cleaved easily by proteases, which leads to HMVWF deficiency and ultimately hemostasis impairment.

Outcomes

Stemming from all the above studies, it can be inferred that the increased shear stress caused by the stenotic aortic valve leads to cleavage of Von Willebrand Factor (VWF). The associated deficiency resulting from the above is proposed to be the reason behind the acquired Von Willebrand deficiency Type 2A. Within several studies, molecular analysis of VWF structure pointed towards a significant reduction in high molecular weight multimers [9-15].

Discussion

Aortic Stenosis (AS) is defined as the narrowing of the aortic valve, caused by a combination of progressive fibrosis and calcification of the matrix. This causes an increase in valve stiffness with a significant reduction in the valve area which increases left ventricular afterload and burden [16]. Aortic valve sclerosis (ASc) occurs in the earliest stages of AS. ASc is associated with calcification and fibrosis without significant left ventricular outflow obstruction [16]. 

In the Western world, the most common form of valvular heart disease is AS. This can be attributed to the incidence of AS in older age. According to the Helsinki Ageing Study, there is 40% to 75% of individuals with detectable valve calcification are between the ages of 65 and 85 years [16]. Heyde syndrome is a multisystemic disorder characterized by the triad of AS, gastrointestinal bleeding, and acquired Von Willebrand disease (AVWD) [17].

Findings From Studies Selected for Review

In a study by Bolliger et al. on a cohort of 60 patients carried out in Switzerland, it was concluded that the identification of altered Von Willebrand factor (VWF) before surgery was not associated with postoperative bleeding. It can be explained by the perioperative release of VWF and rapid recovery of large VWF multimers [9].

Casanato et al. studied a cohort of 41 patients, where they found that within 24 hours of AVR, VWF returned to normal levels. They persisted for a duration of 6 months [10]. Also, it indicates that surgery has an important role to play in the correction of acquired VWF deficiency. These findings support a direct relationship between AS and a decrease in VWF multimers.

A study by Bander et al. on a cohort of 114 patients identified a pathophysiological explanation for the increase in VWF after surgery. ADAMTS-13 declines after surgery which results in high molecular weight multimers of VWF rising in the blood. The ratio of increase in multimers reflects the proportion of decrease which was attributed to the alleviation of AS [11].

Patients with severe AS are particularly susceptible to anemia because they often suffer from acquired coagulopathy (Von Willebrand syndrome type 2A), which increases the risk of bleeding causing anemia which is a common comorbidity in patients with severe AS [12].

In Austria, a case-control study by Kellermair et al. was conducted on 31 patients. It concludes that AVWS in AS patients is not similar to those in Type 2A. Therefore, further studies are required to support the evidence and prevent misclassification [4].This study suggests against Type 2A, however, it still confirms the presence of Acquired Von Willebrand deficiency (AVWS).

Two case reports were reviewed. The first one, by Kapila et al., suggested that platelet adhesion mediated by VWF multimers for hemostasis undergoes degeneration by high shear stress across the stenotic aortic valve, leading to acquired Von Willebrand's disease (Type 2A VWF disease). Surgery alone can result in the cure of this condition (Heyde syndrome) [14].

The second one by Iijima et al. concluded that the shear stress caused by the stenosed aortic valve causes VWF to stretch. Therefore, it causes it to be cleaved easily by proteases which leads to High Molecular weight multimers of Von Willebrand Factor (HMVWF) deficiency leading to hemostasis impairment [15].

Von Willebrand Disease Type2A

A deficiency of high molecular weight VWF multimers leads to a decreased affinity for platelets and sub-endothelium. This manifests as mucosal and cutaneous bleeding (menorrhagia, epistaxis, gastrointestinal bleeding) [18]. Regarding molecular level observations, mutations in the D2 domain, involved in the multimerization process, are found in patients with type 2A.

Von Willebrand Factor Deficiency in AS

At the site of vascular injury, VWF mediates the adhesion of blood platelets [19]. The multimeric structure of VWF confers its hemostatic function, an important feature [20]. In type 2A Von Willebrand disease (VWD), these multimers decrease increasing the risk of severe bleeding [21]. Secreted by endothelial cells, VWF is cleaved in circulation by ADAMTS-13 - a metalloproteinase, especially under high shear stress forces. This reduces large VWF multimers immediately following their secretion [22,23]. This process confers a typical multimer structure to VWF [24,25]. The steady-state distribution of VWF multimers represents a balance between their secretion and their proteolysis into smaller forms. If the balance shifts towards proteolysis, it consequently leads to a loss of large multimers [26]. The proteolytic cleavage by ADAMTS-13 occurs due to the high-shear forces generated by the stenotic valve. This leads to the disappearance of the large VWF multimers. This hypothesis is confirmed by the increased proportion of VWF subunit fragments observed in these patients. This pattern is strictly related to valve stenosis since correcting the cardiac defect improves the proteolytic fragment pattern and restores the large VWF multimers.

However, Kellermair et al., in their study on 31 AS, argued that the multimeric pattern of Acquired Von Willebrand Syndrome (AVWS) in severe AS is not identical to that seen in acquired Von Willebrand syndrome (AVWS) type 2A or VWD type 2A. The article suggests that a lack of electrophoretic standardization and misclassifications contributed to this, and cites more extensive research and analysis for further conclusions [4].

Von Willebrand Factor Multimers Recover Following the Replacement of the Stenotic Aortic Valve

In a study by Bolliger et al. on 60 AS patients, regarding preoperative cases with VWF deficiency, the bleeding risk following surgery was very low. This was explained by the rapid recovery of the multimers and a large release of VWF during the perioperative period [9]. These findings were supported by Bander et al. in their study on 114 patients. The author postulated that the Von Willebrand Ristocetin Cofactor (VVWF: RCo) to Von Willebrand factor antigen (VWF: Ag) ratio, which acts as a surrogate for large VWF multimer activity, increased post aortic valve replacement (AVR) [11]. Kapila et al. mentioned similar findings in their case report on Heyde's syndrome, where they endorsed that valve replacement can cure this condition [14]. A case report by Iijima M et al. concluded that VWF activity improved following aortic valve replacement. However, multimeric structure analysis was not performed. Hence, the authors of this study suggested that molecular analysis could be indispensable [15]. After aortic valve replacement, the large VWF multimers return to normal. If not, it indicates a coexisting inherited VWF exists and therefore patterns will be still abnormal including abnormal von Willebrand Factor Collagen Binding (VWF: CB) ratios that indicate a high risk of bleeding [13].

Based on this published literature, it can be concluded that VWF deficiency caused by loss of multimers occurs in patients with AS. This abnormality predisposes patients to increased bleeding risks. Surgery offers a treatment scope that is evident by the immediate reversal of multimeric structure and a significant increase in the activity of VWF. Furthermore, the reduced risk of bleeding during the post-operative period points toward a similar elucidation [10].

Limitations

The deficiency of VWF multimers is attributed to the high shear stress that has been endorsed repeatedly. Several other studies demonstrate identical improvements during early post-operative periods, cementing the opinion of VWF pathology in AS patients. However, this opposing evidence has been reported in previous literature [5]. The subtype 2A, associated with AS, also has evidence against it. An experimental study with similar conditions mimicking the lesion, contributing laboratory evidence, is unavailable. Consequently, many studies in this interest can be required to determine the impact of this VWF deficiency in AS.

## Conclusions

Von Willebrand disease has been historically associated with bleeding. Here, a much lesser-known condition termed acquired Von Willebrand disease was identified in a patient population with Aortic Stenosis (AS). The high shear stress created by the stenotic valve leads to cleavage of Von Willebrand Factor (VWF) multimers, leading to a state of deficiency. It is a dreaded complication that has largely been associated with arteriovenous malformation which causes gastrointestinal (GI) bleeding, among other factors. Surgical replacement of the valve was proven to reverse this condition. This underlying condition is not commonly known amongst clinicians and physicians. However, peak importance should be given to the underlying risk for other possible significant hemostatic abnormalities. Despite various articles suggesting this relationship, extensive research is indicated to prove a strong causality and develop novel strategies for circumventing associated complications that are not limited to anemia.

## References

[1] Ancona R, Pinto SC (2020). Epidemiology of aortic valve stenosis (AS) and of aortic valve incompetence (AI): is the prevalence of AS/AI similar in different parts of the world?. e-J Cardiol Prac.

[2] Alshuwaykh O, Krier MJ (2018). A case of Heyde syndrome with resolution of gastrointestinal bleeding two weeks after aortic valve replacement. Am J Case Rep.

[3] Peyvandi F, Garagiola I, Baronciani L (2011). Role of von Willebrand factor in the haemostasis. Blood Transfus.

[4] Kellermair J, Ott HW, Spannagl M (2018). Characterization of Von Willebrand factor multimer structure in patients with severe aortic stenosis. Clin Appl Thromb Hemost.

[5] Carrasco E, López R, Rattalino M (2011). Aortic stenosis and acquired von Willebrand disease: lack of association. J Cardiothorac Vasc Anesth.

[6] Page MJ, McKenzie JE, Bossuyt PM (2021). The PRISMA 2020 statement: an updated guideline for reporting systematic reviews. BMJ.

[7] Aromataris E, Munn Z (2020). JBI Manual for Evidence Synthesis - JBI Global Wiki. https://synthesismanual.jbi.global/.

[8] Gagnier JJ, Kienle G, Altman DG, Moher D, Sox H, Riley D (2013). The CARE Guidelines: consensus-based clinical case reporting guideline development. Glob Adv Health Med.

[9] Bolliger D, Dell-Kuster S, Seeberger MD (2012). Impact of loss of high-molecular-weight von Willebrand factor multimers on blood loss after aortic valve replacement. Br J Anaesth.

[10] Casonato A, Sponga S, Pontara E (2011). von Willebrand factor abnormalities in aortic valve stenosis: pathophysiology and impact on bleeding. Thromb Haemost.

[11] Bander J, Elmariah S, Aledort LM (2012). Changes in von Willebrand factor-cleaving protease (ADAMTS-13) in patients with aortic stenosis undergoing valve replacement or balloon valvuloplasty. Thromb Haemost.

[12] Nagao K, Taniguchi T, Morimoto T (2019). Anemia in patients with severe aortic stenosis. Sci Rep.

[13] Casonato A, Galletta E, Cella G, Barbon G, Daidone V (2020). Acquired von Willebrand syndrome hiding inherited von willebrand disease can explain severe bleeding in patients with aortic stenosis. Arterioscler Thromb Vasc Biol.

[14] Kapila A, Chhabra L, Khanna A (2014). Valvular aortic stenosis causing angiodysplasia and acquired von Willebrand's disease: Heyde's syndrome. BMJ Case Rep.

[15] Iijima M, Itoh N, Murase R, Makino Y (2018). A surgical case of aortic stenosis with recurrent gastrointestinal bleeding: Heyde syndrome. Int J Surg Case Rep.

[16] Sverdlov AL, Ngo DT, Chapman MJ, Ali OA, Chirkov YY, Horowitz JD (2011). Pathogenesis of aortic stenosis: not just a matter of wear and tear. Am J Cardiovasc Dis.

[17] Randi AM, Laffan MA, Starke RD (2013). Von Willebrand factor, angiodysplasia and angiogenesis. Mediterr J Hematol Infect Dis.

[18] Fressinaud E, Mazurier C, Meyer D (2002). Molecular genetics of type 2 von Willebrand disease. Int J Hematol.

[19] Sadler JE (2005). von Willebrand factor: two sides of a coin. J Thromb Haemost.

[20] Gralnick HR, Williams SB, Morisato DK (1981). Effect of multimeric structure of the factor VIII/von Willebrand factor protein on binding to platelets. Blood.

[21] Lyons SE, Bruck ME, Bowie EJ, Ginsburg D (1992). Impaired intracellular transport produced by a subset of type IIA von Willebrand disease mutations. J Biol Chem.

[22] Tsai HM, Sussman II, Nagel RL (1994). Shear stress enhances the proteolysis of von Willebrand factor in normal plasma. Blood.

[23] Dong JF, Moake JL, Bernardo A (2003). ADAMTS-13 metalloprotease interacts with the endothelial cell-derived ultra-large von Willebrand factor. J Biol Chem.

[24] Dent JA, Galbusera M, Ruggeri ZM (1991). Heterogeneity of plasma von Willebrand factor multimers resulting from proteolysis of the constituent subunit. J Clin Invest.

[25] Furlan M, Robles R, Affolter D, Meyer D, Baillod P, Lämmle B (1993). Triplet structure of von Willebrand factor reflects proteolytic degradation of high molecular weight multimers. Proc Natl Acad Sci U S A.

[26] Tsai HM, Sussman II, Ginsburg D, Lankhof H, Sixma JJ, Nagel RL (1997). Proteolytic cleavage of recombinant type 2A von Willebrand factor mutants R834W and R834Q: inhibition by doxycycline and by monoclonal antibody VP-1. Blood.

